# The impact of *CYP2C19* genotype on phenoconversion by concomitant medication

**DOI:** 10.3389/fphar.2023.1201906

**Published:** 2023-06-08

**Authors:** Laura M. de Jong, Soukayna Boussallami, Elena Sánchez-López, Martin Giera, Maarten E. Tushuizen, Menno Hoekstra, Lukas J. A. C. Hawinkels, Robert Rissmann, Jesse J. Swen, Martijn L. Manson

**Affiliations:** ^1^ Division of Systems Pharmacology and Pharmacy, Leiden Academic Centre for Drug Research, Leiden, Netherlands; ^2^ Center for Proteomics and Metabolomics, Leiden University Medical Center, Leiden, Netherlands; ^3^ Department of Gastroenterology and Hepatology, Leiden University Medical Center, Leiden, Netherlands; ^4^ Centre for Human Drug Research, Leiden, Netherlands; ^5^ Division of BioTherapeutics, Leiden Academic Centre for Drug Research, Leiden, Netherlands; ^6^ Department of Dermatology, Leiden University Medical Center, Leiden, Netherlands; ^7^ Department of Clinical Pharmacy and Toxicology, Leiden University Medical Center, Leiden, Netherlands

**Keywords:** phenoconversion, pharmacogenetics, drug-drug interactions, drug-drug-gene interactions, drug metabolism, CYP2C19

## Abstract

**Introduction:** Pharmacogenetics-informed drug prescribing is increasingly applied in clinical practice. Typically, drug metabolizing phenotypes are determined based on genetic test results, whereupon dosage or drugs are adjusted. Drug-drug-interactions (DDIs) caused by concomitant medication can however cause mismatches between predicted and observed phenotypes (phenoconversion). Here we investigated the impact of *CYP2C19* genotype on the outcome of CYP2C19-dependent DDIs in human liver microsomes.

**Methods:** Liver samples from 40 patients were included, and genotyped for CYP2C19*2, *3 and *17 variants. S-mephenytoin metabolism in microsomal fractions was used as proxy for CYP2C19 activity, and concordance between genotype-predicted and observed CYP2C19 phenotype was examined. Individual microsomes were subsequently co-exposed to fluvoxamine, voriconazole, omeprazole or pantoprazole to simulate DDIs.

**Results:** Maximal CYP2C19 activity (V_max_) in genotype-predicted intermediate metabolizers (IMs; *1/*2 or *2/*17), rapid metabolizers (RMs; *1/*17) and ultrarapid metabolizers (UMs; *17/*17) was not different from V_max_ of predicted normal metabolizers (NMs; *1/*1). Conversely, CYP2C19*2/*2 genotyped-donors exhibited V_max_ rates ∼9% of NMs, confirming the genotype-predicted poor metabolizer (PM) phenotype. Categorizing CYP2C19 activity, we found a 40% concordance between genetically-predicted CYP2C19 phenotypes and measured phenotypes, indicating substantial phenoconversion. Eight patients (20%) exhibited CYP2C19 IM/PM phenotypes that were not predicted by their CYP2C19 genotype, of which six could be linked to the presence of diabetes or liver disease. In subsequent DDI experiments, CYP2C19 activity was inhibited by omeprazole (−37% ± 8%), voriconazole (−59% ± 4%) and fluvoxamine (−85% ± 2%), but not by pantoprazole (−2 ± 4%). The strength of CYP2C19 inhibitors remained unaffected by *CYP2C19* genotype, as similar percental declines in CYP2C19 activity and comparable metabolism-dependent inhibitory constants (K_inact_/K_I_) of omeprazole were observed between CYP2C19 genotypes. However, the consequences of CYP2C19 inhibitor-mediated phenoconversion were different between *CYP2C19* genotypes. In example, voriconazole converted 50% of *1/*1 donors to a IM/PM phenotype, but only 14% of *1/*17 donors. Fluvoxamine converted all donors to phenotypic IMs/PMs, but *1/*17 (14%) were less likely to become PMs than *1/*1 (50%) or *1/*2 and *2/*17 (57%).

**Conclusion:** This study suggests that the differential outcome of CYP2C19-mediated DDIs between genotypes are primarily dictated by basal CYP2C19 activity, that may in part be predicted by *CYP2C19* genotype but likely also depends on disease-related factors.

## Introduction

Pharmacogenetics aims to increase patient safety and drug efficacy by tailoring drug treatment to an individual’s genetic profile. Based on this genetic profile, patients can be categorized into drug metabolizing phenotypes which subsequently can be used for selecting the right drug and optimal dose. Therapeutic guidance for actionable drug-gene interactions (DGIs) have been developed by the Clinical Pharmacogenetics Implementation Consortium (CPIC) and the Dutch Pharmacogenetic Work Group (DPWG) for over 75 drugs (Swen et al., 2011; CPIC, 2023). However, a common problem encountered using drug metabolizing phenotypes is that a patient’s genetically-predicted phenotype can deviate from its actual metabolizer status—a phenomenon called phenoconversion (Shah and Smith, 2015; Klomp et al., 2020).

Non-genetic factors that skew this genotype-based prediction include inflammatory or liver diseases as well as drug-drug interactions (DDIs) caused by concomitant medication use (Shah and Smith, 2015). The individual impact of genetic polymorphisms and DDIs on pharmacokinetics of drugs has been vastly investigated. However, the interplay between pharmacogenetics and DDIs that may result in drug-drug-gene interactions (DDGIs) is not yet taken into account in clinical practice. Importantly, DDGIs account for up to 20% of total major or substantial drug interactions and are thus a clinical concern (Verbeurgt et al., 2014; Hocum et al., 2016).

Numerous studies demonstrate that a patient’s genotype determines the clinical relevance of a DDGI (Bahar et al., 2017). For example, Storelli *et al.* showed that the presence of one nonfunctional *CYP2D6* allele increases the risk of phenoconversion to a poor metabolizer (PM) status in the presence of a CYP2D6 inhibitor (Storelli et al., 2018). This suggests that the occurrence of DDIs in patients with reduced enzyme functionality at baseline creates a higher susceptibility for phenoconversion towards an actionable genotype. In contrast, PMs are not considered prone to DDIs involving the same enzyme, as these individuals already exhibit null enzymatic activity at baseline. Considering the importance of DDI-induced phenoconversion, CPIC guidelines suggest that the concomitant use of CYP2D6 inhibitors should be taken into account for calculating the genotype-based activity score (Crews et al., 2021).

The *CYP2C19* gene is highly polymorphic and responsible for metabolism of frequently prescribed proton-pump inhibitors (PPIs) and other commonly used drugs including clopidogrel and antidepressants. A large proportion of CYP2C19-related drugs acts as CYP2C19 inhibitors, for which concomitant use may result in DDIs. As a consequence, concomitant medication use may commonly lead to phenoconversion of CYP2C19-mediated metabolism. For instance, when considering phenoconversion caused by DDGIs, the CYP2C19 PM phenotype was found 5-fold more frequently than expected based on genotype alone in a group of 2905 patients (Mostafa et al., 2019). Consequently, the predicted phenotype based on genotype solely could be erroneous when concomitant use of CYP2C19 inhibitors is not contemplated while predicting CYP2C19 phenotype. However, phenoconversion rates for CYP2C19-mediated drug metabolism following treatment with an inhibitor have not been determined due to sparse availability of data to help predict the drug metabolizing phenotype after inhibitor use.

To ultimately provide concise DDGI recommendations that combine knowledge on pharmacogenetics and concomitant medication use, it is important to gain a quantitative understanding of the phenoconversion that occurs after co-administration of an inhibitor of the same enzyme. To this end, we aimed to quantify to what extent *CYP2C19* polymorphisms can impact the outcome of a DDI with various CYP2C19 inhibitors in human liver microsomes. Firstly, we set out to assess the genotype-phenotype discordance in this cohort and link this to known phenoconversion risk factors. We then investigated whether the intrinsic inhibitory activity of the most prescribed PPI and CYP2C19 inhibitor omeprazole was affected by the *CYP2C19* genotype. Lastly, we quantified phenoconversion after co-administration of various clinically relevant CYP2C19 inhibitors.

## Materials and methods

### Human liver samples

Macroscopically healthy liver samples from 40 patients with colorectal cancer derived liver metastasis were retrieved from the gastroenterology biobank at the Leiden University Medical Center (LUMC, Leiden, Netherlands). Fresh tissue samples were obtained directly after surgery, and macroscopically healthy liver tissues distant from the metastasis (at tumor free resection margins) were collected, snap frozen end stored at −80°C until use. The collection and use of these samples was approved by the Medical Ethics Committee of Leiden Den Haag Delft, Netherlands through protocol B21.072 entitled “The modulating potential of CYP450 genetic variability on phenoconversion by concomitant medication.”

### Genotyping

Genomic DNA from the human liver samples was extracted using the NucleoSpin Tissue mini kit from Macherey-Nagel (Hoerdt, France). The *CYP2C19* variant alleles CYP2C19*2 (*NC_000010.11: g.94781859G>A*), CYP2C19*3 (NC_000010.11: g.94780653G>A), and CYP2C19*17 (NC_000010.11: g.94761900C>T) were analyzed using pre-designed TaqMan-based real-time polymerase chain reaction (PCR) assays, with probes obtained from ThermoFisher. The Quantstudio and ViiA7 systems were employed for analysis. All genotyping was conducted following standard protocols used in routine diagnostics, in an ISO-15189 certified laboratory. The variants were checked for Hardy-Weinberg equilibrium. Predicted phenotypes were assigned using conventional methods based on translation tables from CPIC and DPWG (PharmGKB, 2023).

### RNA preparation and real time-qPCR

Liver RNA was isolated using the RNeasy Mini Kit (Qiagen, Hilden, Germany) according to the manufacturer’s instructions. Concentration and purity of RNA was subsequently measured using a NanoDrop 3300 (Thermo Scientific, Wilmington, US). RNA was reverse-transcribed into cDNA using a RevertAid H Minus First Strand cDNA Synthesis kit (Thermo Scientific, Wilmington, US) according to the instructions provided. RT-qPCR analysis was performed using a QuantStudio™ 6 Flex System.

All PCR primers were designed in-house and subsequently checked for amplification efficiency through a serial dilution of cDNA where 90%–110% efficiency was desired (Supplementary Table S1). A CYP2C19 primer targeting exon 9 was designed to amplify total CYP2C19 mRNA. As this primer does not distinguish between mRNA encoding for functional or non-functional CYP2C19 protein, an additional exon-spanning primer pair was designed that could predominantly detect functional mRNA. This was achieved through a reverse primer binding within the first 40 basepairs of exon 5, as this region is deleted in CYP2C19*2 carriers and the most commonly observed variant linked to the formation of non-functional CYP2C19 protein (Chaudhry et al., 2015).

Relative mRNA levels were calculated using the comparative Ct method and normalized to the geometric mean of the housekeeping genes Ribosomal Protein Lateral Stalk Subunit P0 *(RPLP0)* and RNA Polymerase II, I and III Subunit L *(POLR2L)*, which were determined as the most stable endogenous controls through GNOrm software analysis (Vandesompele et al., 2002).

### Liver microsomal preparations

Human liver microsomes were prepared from obtained liver resections with the aid of a microsome isolation kit from Sigma-Aldrich (St. Louis, MO, United States). Total protein concentrations were determined in triplicate with the BCA protein assay (Pierce, Rockford, IL, United States). Aliquots of the final microsomal suspension were stored at −80°C. The microsomal protein per gram of liver (MPPGL, mg/g) was calculated by dividing the microsomal protein yield by the liver weight input and was on average 7.4 ± 2.0 mg/g in this cohort. Individual microsomal preparations were used for all experiments except for the experiment in which inhibitory parameters of omeprazole were determined. In these omeprazole-related experiments, genotype-matched microsome pools where generated by pooling an equal amount of microsomal protein from either 8 (*1/*17), 16 (*1/*1) or 10 (*1/*2 or *2/*17) donors.

### CYP2C19 activity assays in microsomes

#### Kinetic analysis of CYP2C19 dependent S-mephenytoin hydroxylation

Various concentrations of S-mephenytoin (1–400 µM) were incubated with individual genotyped human liver microsomes (final protein concentration: 0.03 mg/mL) in 200 µL incubation mixtures containing 0.05 mM potassium phosphate buffer (pH 7.4) with MgCl_2_ (3 mM), EDTA (1 mM), NADP (1 mM), glucose-6-phosphate (5 mM) and glucose-6-phosphate dehydrogenase (1 unit/mL). Incubations were performed in duplicate in Protein LoBind^®^ Tubes (Eppendorf, Hamburg, Germany). After 30 min, reactions were terminated by the addition of equal volumes of ice-cold acetonitrile containing the internal standard 4′-hydroxymephenytoin-d_3_ (20 ng/mL). Insoluble protein was precipitated by centrifugation (10,000 × g for 5 min at 4°C), and supernatant was diluted 2.5 times in LC-MS quality water before 4′-hydroxymephenytoin concentration measurements. A validated liquid chromatography-tandem mass spectrometry (LC-MS/MS) assay was used to quantify 4′-hydroxymephenytoin (see “Quantification of 4′-hydroxymephenytoin by LC-MS/MS, Supplementary Material”).

#### Determination of kinetic parameters

Maximal velocity of S-mephenytoin 4′-hydroxylation (V_max_) and affinity (K_m_) values were obtained for each individual donor by fitting individual data to the Michaelis-Menten equation: $V=\frac{V_{max}\left[S\right]}{K_{m}\left[S\right]}$ in Graphpad Prism 9 (Graphpad Software, San Diego, CA), where *V* represents the initial metabolism rate of S-mephenytoin (pmol/min/mg protein) and [S] represents the S-mephenytoin substrate concentration (µM). No Michaelis-Menten curve fitting was done for donors with non-saturable product formation kinetics. For these donors, V_max_ values were estimated by means of simple linear regression. K_m_ values were only determined when S-mephenytoin 4′-hydroxylation followed Michaelis-Menten kinetics. To analyze the kinetic parameters for S-mephenytoin 4′-hydroxylation across donors with the same genotype, non-linear least-squares analysis in Graphpad Prism was done without restrictions.

#### Determination of basal phenoconversion in cohort

*CYP2C19* genotypes were first used to predict the drug metabolizing activity of donors classified into the phenotype categories: ultrarapid metabolizer (UM), rapid metabolizer (RM), normal metabolizer (NM), intermediate metabolizer (IM) and poor metabolizer (PM), according to CPIC guidelines (PharmGKB, 2023). Secondly, cut-off values for the metabolic activity of phenotype groups were defined based on the study by Kiss *et al.*, in which S-mephenytoin hydroxylation at a saturating substrate concentration was determined in genotyped liver microsomes of 114 donors (Kiss et al., 2018). Since Kiss *et al.* did not define a RM group, boundaries between NMs and RMs were determined using the same method and thus based on the median S-mephenytoin hydroxylation activity in 24 donors. Hence, cut-off values between the phenotypic groups PM/IMs, IMs/NMs, NMs/RMs and RMs/UMs were set in this study at 8, 23, 58, and 75 pmol/min/mg protein respectively.

The observed maximal S-mephenytoin hydroxylation activity in individual donors was then compared to the expected activity for these donors based on their genotype-predicted phenotype. Concordance/non-concordance between measured and genotype-predicted hydroxylation activity was determined for every individual donor to indicate basal phenoconversion.

#### Determination of inhibitor-induced phenoconversion

##### Inhibitor concentrations

To simulate the outcome of DDIs for different *CYP2C19* genotypes, individual microsomal fractions were co-exposed to clinically relevant concentrations of the CYP2C19 inhibitors fluvoxamine, voriconazole, omeprazole or pantoprazole. Concentrations were based on the calculated unbound maximum hepatic inlet concentration in plasma (I_in,max,u_), which incorporates both the drug entering the liver from the systemic circulation as well as the drug entering the liver from the gut via the hepatic portal vein following the equation: (Parkinson, 2019):

$$I_{in,\max,u}={Fu}_{p}\;\left(Plasma\;I_{\max}+\frac{\left(\frac{Dose*Fa*Fg*Ka}{Qh}\right)}{Rb}\right)$$

where Fu_p_ is the fraction unbound in plasma, Plasma I_max_ represents the total systemic C_max_ in plasma, Dose is the oral dose, Fa*Fg represent the fraction of drug absorbed from the gastrointestinal tract into the hepatic portal blood, Ka is the rate of absorption of drug from the intestine, Qh is the hepatic blood flow and Rb the drug concentration in blood to the drug concentration in plasma.

Input parameters were retrieved from literature and are described in Table 1, as well as the final calculated I_in,max,u_ used in this assay. The calculation of the I_in,max,u_ was based on the clinically standard starting dose for all inhibitors. The Qh was assumed to be 1,62 L/min (as used by all regulatory agencies). Input plasma I_max_ values are detailed in the Supplementary Material under “Calculating the unbound maximum hepatic inlet concentration”.

**TABLE 1:** Input parameters for calculating the unbound maximum hepatic inlet concentration in plasma (I_in,max,u_). In the absence of experimentally determined values, the Ka was assumed to be 0.1 min^−1^, and the Fa*Fg and Rb were assumed to be 1 (Parkinson, 2019).

	Dose (mg)	Dose (µmol)	Mean plasma I_max_ (µM)*	Ka (min^−1^)	References Ka	Rb	References Rb	Fraction unbound in plasma (Fu_p_)**	I_in,max,u_ (µM)
Fluvoxamine	100	314.0	0.3	0.020	Iga (2015)	1.0		0.25	1.0
Omeprazole	40	115.8	3.3	0.100	Ogilvie et al. (2011)	0.6	Ogilvie et al. (2011)	0.05	0.8
Voriconazole	200	572.6	7.3	0.012	Shi et al. (2019)	2.1	Grensemann et al. (2021)	0.42	3.9
Pantoprazole	40	104.3	6.5	0.018	Gawroñska-Szklarz et al. (2012)	1.0		0.02	0.2

*References for mean plasma I_max_ levels can be found in the Supplementary Table S3.

**Fraction unbound was derived from the drug prescribing information.

##### Incubations with inhibitors

From the 40 donors, 10 donors had a maximum rate of formation lower than 10 pmol/min/mg protein in the absence of inhibitors, which corresponds to a PM phenotype. These donors were therefore excluded in subsequent experiments in which the consequences of the different CYP2C19 inhibitors were determined. To assess the direct inhibition of CYP2C19 by fluvoxamine, voriconazole and pantoprazole for the 30 individual donors, the selected concentrations of inhibitors were incubated with 30 µM of S-mephenytoin (frequently reported K_m_ value), microsomes (0.03 mg/mL) and the NADPH generating system described above in 0.05 mM phosphate buffer (pH = 7.4) for 7 min. Incubations without inhibitor served as control. Omeprazole is a metabolism-dependent inhibitor (MDI) of CYP2C19, meaning that the formation of omeprazole metabolites increases the inhibitory potency of omeprazole over time (Ogilvie et al., 2011). To simulate the MDI of CYP2C19 by omeprazole, omeprazole was pre-incubated at 37°C with NADPH-fortified microsomes for 40 min. After the pre-incubation, S-mephenytoin (30 μM, final) was supplemented and the incubation time was continued for 7 min to measure residual CYP2C19 activity. Incubations without omeprazole but with 40 min pre-incubation served as control.

##### Cut-off values phenotype groups

Published thresholds for defining CYP2C19 phenotype categories are only available at formation rates determined with maximal substrate stimulation (Kiss et al., 2018). In order to investigate DDI-induced phenoconversion, the rate of formation for individual donors was determined at S-mephenytoin concentration of 30 µM. A calculated scaling factor (activity at 400 µM/activity at 30 µM) was used to transform the phenotype cut-off thresholds used at maximum substate formation. Accordingly, thresholds between the phenotypic groups PM/IMs, IMs/NMs, NMs/RMs and RMs/UMs were 5, 14, 40 and 53 pmol/min/mg protein.

##### K_I_ and K_inact_ determinations for omeprazole

K_I_ (inhibitor concentration that supports half the maximal rate of inactivation) and K_inact_ (maximal rate of enzyme inactivation) parameters were determined as described by Ogilvie *et al.* (Ogilvie et al., 2011), using the non-dilution method (Parkinson et al., 2011). In order to determine K_I_ and K_inact_ values for the inactivation of CYP2C19 by omeprazole, genotype-pooled microsomes were pre-incubated with various concentrations of omeprazole (1–30 µM) for 0–30 min at 37°C. After pre-incubation, S-mephenytoin (30 µM) was added and residual CYP2C19 activity was determined as described under “Kinetic analysis of CYP2C19 dependent S-mephenytoin hydroxylation.” K_I_ and K_inact_ parameters were determined using non-linear regression in Graphpad Prism 9.

### Chemicals and reagents

S-mephenytoin, 4′-hydroxymephenytoin, 4′-hydroxymephenytoin-d_3_, voriconazole and omeprazole were purchased from LGC (Wesel, Germany). Fluvoxamine maleate was purchased from Tocris (Bristol, United Kingdom). Pantoprazole sodium, nicotinamide adenine dinucleotide phosphate (NADP), glucose-6-phosphate and glucose-6-phosphate dehydrogenase from baker’s yeast (*S. cerevisiae*) were purchased from Sigma-Aldrich. Acetonitrile, methanol, water and formic acid of LC-MS grade were obtained from Merk (Darmstadt, Germany).

### Statistical analysis

For data which showed no normal distribution based on the Shapiro-Wilk test of normality and QQ-plots, the Kruskal–Wallis test was performed followed by a Dunnett’s multiple comparison test to compare genotype-groups. For normally distributed data, the one-way ANOVA followed by a Dunnett’s multiple comparison test was used. Correlation analysis were performed with the non-parametric Spearman test. A *p*-value of <0.05 was considered to be statistically significant.

## Results

### Patient characteristics

A total of 40 liver samples from 15 female, 23 male and 2 donors of unknown sex were included in the study. The patient characteristics are summarized in Table 2. Complete information on age, body mass index (BMI), comorbidities and concomitant medication use at the time of surgery was not always available from the medical records. Of the donors, 12.5% suffered from an additional liver disease, 17.5% from a chronic inflammatory disease, 12.5% patients had diabetes mellitus and 5% of patients used CYP2C19 inhibitors before surgery.

**TABLE 2:** Population characteristics of the cohort.

	Mean (N)	Range
Age (years)	62.6 (38)	42–87
BMI (kg/m^2^)	26.9 (28)	18–37

	N	%
Female	15	37.5
Male	23	57.5
Unknown	2	5.0
Liver disease		
Cirrhosis	1	2.5
Cholangitis	2	5.0
Choledocholithiasis	1	2.5
Liver abscess	1	2.5
None	30	75.0
Unknown	5	12.5
Inflammatory disease		
Skin	2	5.0
Lung	4	10.0
Joins	1	2.5
None	29	72.5
Unknown	5	12.5
Diabetes mellitus		
Present	5	12.5
Not present	30	75.0
Unknown	5	12.5
Drug use before operation		
CYP2C19 inhibitor	2	5.0
CYP2C19 inducer	0	0.0
None	20	50.0
Unknown	18	45.0

### Genotyping

Liver donors were genotyped for *CYP2C19* variants *1, *2, *3, and *17. All allele variants were consistent with Hardy-Weinberg equilibrium (*2: x^2^ = 3.2, *p* = 0.07, *17: x^2^ = 0.4, *p* = 0.54, *1: x^2^ = 2.05, *p* = 0.15). CYP2C19*3 was not detected in the study samples. *CYP2C19* genotype frequencies and predicted phenotypes are summarized in Table 3. Expected genotype frequencies were in concordance with reported frequencies in the PharmGKB database for Europeans (PharmGKB, 2023).

**TABLE 3:** Genotype distribution and frequency in this study population and corresponding mean kinetic parameters (V_max_ and K_m_) for CYP2C19-catalyzed S-mephenytoin metabolism per CYP2C19 genotype. Kinetic parameters were obtained from the data presented in Figure 1A.*p < 0.05, significantly different from kinetic parameter in CYP2C19*1/*1 donors.

CYP2C19 genotype	Observed frequency N (%)	Expected frequency^#^ (%)	Genotype-predicted phenotype^&^	V_max_ (pmol/min/mg protein)	K_m_ (µM)
				Mean ± SD	Mean ± SD
*1/*1	16 (40.0)	39.1	NM	50.2 ± 36.5	18.4 ± 4.8
*1/*2	7 (17.5)	18.3	IM	32.3 ± 28.1	21.2 ± 5.5
*2/*17	3 (7.5)	6.3	IM	42.2 ± 37.5	23.0 ± 7.4
*2/*2	4 (10.0)	2.2	PM	4.3 ± 2.9*	-
*1/*17	8 (20.0)	26.7	RM	60.4 ± 32.2	18.8 ± 3.9
*17/*17	2 (5.0)	4.6	UM	28.1 ± 6.1	33.4 ± 8.4
total	40 (100)				

^#^Based on genotype frequencies for Europeans in PharmGKB.

^&^Translation based on PharmGKB database (PharmGKB, 2023). NM, normal metabolizer; IM, intermediate metabolizer; PM, poor metabolizer; RM, rapid metabolizer; UM, ultrarapid metabolizer.

### Impact of genotype on CYP2C19-mediated metabolism of S-mephenytoin

**FIGURE 1:**
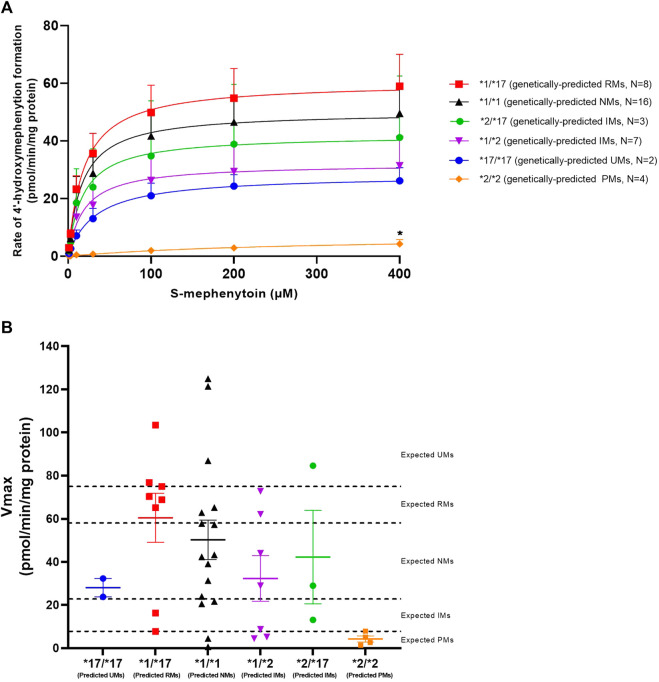
Kinetic analysis of CYP2C19-mediated S-mephenytoin metabolism in genotype-matched donors. **(A)** Mean velocities +SEM at each substrate concentration are shown. Between genotype-group comparisons of maximal 4′-hydroxymephenytoin formation was done using a Kruskal–Wallis test with a Dunn’s multiple comparisons test to *1/*1. *p < 0.05. **(B)** Maximal measured CYP2C19 activity (symbols) *versus* genetically-predicted maximal CYP2C19 activities from literature (dotted lines) in subjects with different CYP2C19 genotypes. Cut-off values for CYP2C19 phenotype groups are based on Kiss *et al.* (Kiss et al., 2018). Means per genotype +SEM are shown.

CYP2C19 activity was measured in all genotyped liver microsomes using S-mephenytoin as a probe substrate. Formation of 4′-hydroxymephenytoin was saturable for all investigated genotypes, with the exception of the *2/*2 genotype (Figure 1A). Michaelis-Menten parameters were obtained from the kinetic analysis of individual donors (Table 3). Mean maximal velocity rates (V_max_) were comparable to S-mephenytoin 4′-hydroxylation activities in microsomes published by Shirasaka *et al.* (Shirasaka et al., 2016). Compared with the CYP2C19*1/*1 genotype, donors with the CYP2C19*2/*2 genotype exhibited decreased V_max_ values (∼9% of *1/*1, *p* = 0.04). V_max_ values of all other genotypes did not differ from that of *1/*1. CYP2C19 substrate affinities (K_m_) were, as expected, not different between genotype groups. Importantly, K_m_ values were comparable to published microsomal affinity values of S-mephenytoin for CYP2C19 (Shirasaka et al., 2016).

To investigate basal phenoconversion, genotype-predicted drug metabolizing phenotypes (PM, IM, NM, RM or UM) were compared to the observed activities of individual donors (Figure 1B). All genetically-predicted PMs indeed showed a PM phenotype, indicative of a complete loss of functional CYP2C19 activity. However, the 4′-hydroxylation activity of six other donors also corresponded to a PM phenotype. In contrast, five donors showed an UM phenotype despite not having two increased function alleles (*17). Altogether, a relatively low concordance (40%) was observed between measured CYP2C19 metabolizing phenotype for the donors within this study and literature based genotype-predicted phenotypes, suggesting the occurrence of phenoconversion in absence of concomitant medication use.

### Correlation between CYP2C19 mRNA levels and metabolic activity

CYP2C19 enzyme activity is both affected by genetic polymorphisms as well as disease-related factors including inflammation and chronic liver disease (Zanger and Schwab, 2013). We therefore set out to assess the predictive relationship of CYP2C19 mRNA expression levels for CYP2C19 activity, and link demographic variables from this cohort to metabolic activity to find explanations for the observed discrepancy between genotype-predicted activity and measured metabolizing phenotype.

**FIGURE 2:**
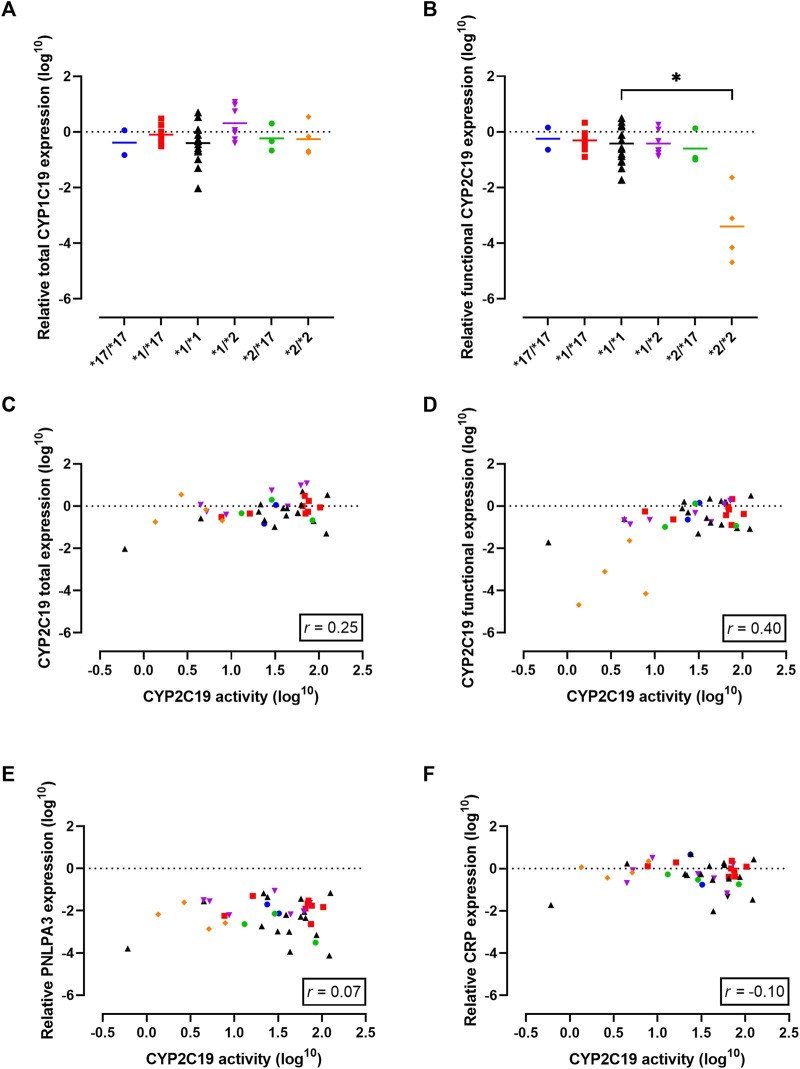
Gene expression analysis in the cohort to investigate the observed discrepancy between genotype-predicted CYP2C19 activity and measured CYP2C19 activity. **(A)** Total CYP2C19 mRNA expression stratified per genotype. Individual values +means per genotype are presented. **(B)** Levels of mRNA that lead to functional CYP2C19 protein stratified per genotype. Individual values +means are presented. **(C)** Correlation between CYP2C19 mRNA and enzyme activity for total mRNA levels and **(D)** levels of mRNA that lead to functional CYP2C19 protein. **(E)** Correlation between CYP2C19 enzyme activity and known regulators of CYP2C19 activity: liver disease (PNPLA3) and **(F)** inflammation (CRP). Blue circles represent *17/*17 donors, red squares represent *1/*17 donors, black triangles represent *1/*1 donors, purple triangles represent *1/*2 donors, green circles represent *2/*17 donors and orange diamond represent *2/*2 donors. Spearman correlation (r) was calculated using GraphPad Prism 9.

First, total CYP2C19 mRNA transcriptional levels for the different genotypes were examined. The different genotype groups did not exhibit differences in total CYP2C19 mRNA expression levels (Figure 2A). One significant limitation of mRNA expression studies is that the functional consequences of the mRNA produced are often not considered. In the case of CYP2C19, the presence of the CYP2C19*2 allele is linked to splicing defects in mRNA production and the formation of inactive protein (Chaudhry et al., 2015). To address this limitation, we utilized a primer-pair that primarily detects functional mRNA rather than CYP2C19*2 mRNA. Indeed, functional CYP2C19 expression levels were dramatically reduced in the *2/*2 genotype as compared to the *1/*1 genotype (*p* = 0.01, Figure 2B). Mean functional CYP2C19 expression levels followed the rank order of *17/*17, *1/*17, *1/*1, *1/*2, *2/*17, and was lowest for *2/*2, as would be expected based on allele functionality.

Next, mRNA expression levels were correlated to measured CYP2C19 metabolizing activities to investigate a potential predictive relationship. Total CYP2C19 expression levels did not correlate with CYP2C19 activity (*r* = 0.25, *p* = 0.12, Figure 2C). In contrast, the activity level of CYP2C19 was positively correlated with functional CYP2C19 mRNA levels (*r* = 0.40, *p* = 0.01, Figure 2D), suggesting transcriptional regulation may in part explain the differences in enzyme activity between the genotype groups. It should however be noted that this increased positive correlation as compared to total mRNA levels was mainly driven by PM donors.

### Influence of disease-related factors and concomitant medication on CYP2C19 metabolic activity

Liver disease is a non-genetic factor shown to alter CYP450 activity (Ohnishi et al., 2005; Duthaler et al., 2022). PNPLA3 is an established genetic marker of progressive liver disease (Dubuquoy et al., 2013), but PNPLA3 mRNA expression did not correlate to CYP2C19 activity in this cohort (*r* = 0.07*, p* = 0.68, Figure 2E). Among the five patients with confirmed liver disease, the presence of cirrhosis, cholangitis or liver abscess was associated with lower CYP2C19 activity compared to what’s expected based on genotype. Importantly, this included two genetically-predicted RMs that phenoconverted to an IM or PM phenotype, and one *1/*1 donor that converted to a PM phenotype. Diabetes mellitus is recently identified as a modifying factor of CYP2C19 activity, with patients displaying mean reduced activity of ∼50% (Gravel et al., 2019). In our cohort, 5 patients suffered from diabetes mellitus of which one was genetically-predicted PM. For the other four donors, three of them showed phenoconversion to a PM phenotype. Inflammation is another non-genetic factors altering CYP2C19 activity (de Jong et al., 2020). Overall, there was no correlation between mRNA levels of CRP, a measure of inflammation, and CYP2C19 activity (*r* = −0.10, *p* = 0.53, Figure 2F). In line, although 17.5% of patients in this cohort suffered from a (systemic) inflammatory disease, not all of them displayed phenoconversion.

The use of concurrent medication can also lead to phenoconversion, as this can result in induced expression or inhibition of drug metabolizing enzymes (Klomp et al., 2020). Prior to surgery, two patients were on CYP2C19 inhibitor therapy. No phenoconversion was evident for the patient on pantoprazole, in line with its classification as a weak inhibitor. The second patient exhibited a PM phenotype despite their *1/*17 genotype. The underlying cause of this phenoconversion could be dual, as this patient was using esomeprazole before surgery and suffered from the comorbidity cholangitis. It is crucial to note that unlike CYP induction, the inhibition in liver microsomes caused by clinically administered CYP2C19 inhibitors is less probable to persist due to the necessary washing steps in the liver microsome isolation and the reversible nature of CYP inhibition.

### Genotype-dependent impact of drug-drug interactions

**FIGURE 3:**
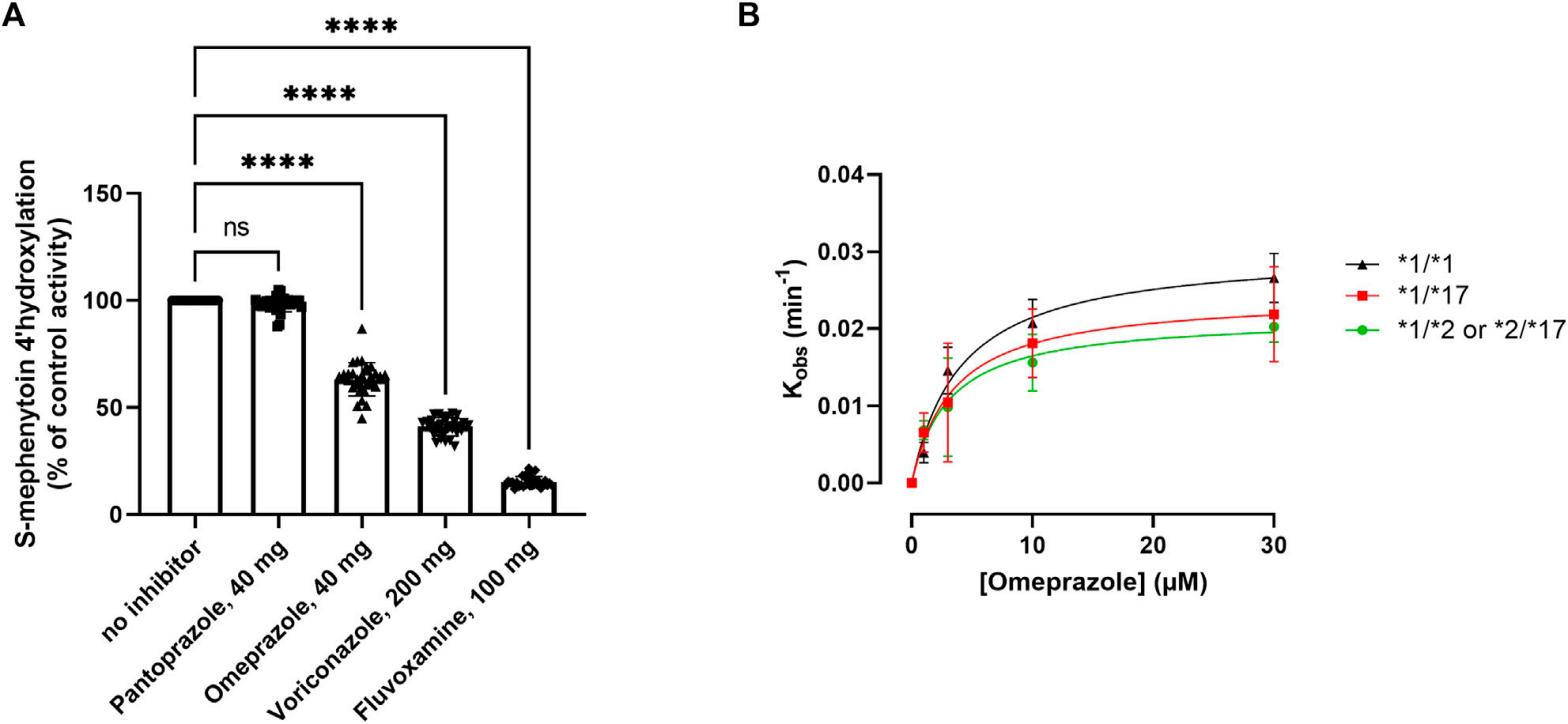
Kinetic analysis of the impact of various CYP2C19 inhibitors on CYP2C19 activity and inactivation. **(A)** Impact of selected CYP2C19 inhibitors on CYP2C19 activity for all included donors. Donors that were phenotypically PMs at baseline were excluded for treatment with inhibitors. 4′-hydroxylation activity is shown as compared to control, where omeprazole is matched to its own time-dependent control. A one way ANOVA with matching was done to test the impact of the inhibitors; ****p < 0.0001. **(B)** K_inact_ and K_I_ determinations for the MDI of CYP2C19 by omeprazole for the various genotype groups. The values of the apparent inactivation rate constant (K_obs_) at each concentration of omeprazole are obtained from the slopes of the initial rates of inactivation (Supplementary Figure S1). Individual data points represent the average of three separate experiments ±SD.

The main objective of this study was to assess the occurrence of phenoconversion in various *CYP2C19* genotype groups following administration of either a strong (fluvoxamine), moderate (omeprazole or voriconazole) or weak (pantoprazole) inhibitor of CYP2C19, and thereby quantify to which phenotype they switch. On a group-level, CYP2C19 activity was inhibited (*p* < 0.0001) by omeprazole (−37% ± 8%), voriconazole (−59% ± 4%) and fluvoxamine (−85% ± 2%), but not by pantoprazole (−2 ± 4%) (Figure 3A). This percental decrease in activity was independent of *CYP2C19* genotype (Supplementary Figure S2), indicating that inhibitor strength is not affected by *CYP2C19* genotype.

Omeprazole is a metabolism-dependent inhibitor (MDI) of CYP2C19, meaning that biotransformation of the substrate into its active metabolites contributes to the inhibitory potency of the drug. Since genotype impacts the degree of metabolite formation, we investigated whether the inhibitory potency of omeprazole would be affected by *CYP2C19* genotype. The inhibitory constants K_inact_ (the first order rate constant of CYP2C19 inactivation) and K_I_ (concentration of omeprazole supporting half-maximal rate of CYP2C19 inactivation) were determined in genotype-matched donor pools (Figure 3B). Genotype-matched donor pools were either a pool of donors with two wild type alleles (*1), one non-functional allele (*2) or one gain-of-function allele (*17). *17/*17 donors were excluded due to their already low activities at baseline (basal phenoconversion). For the various genotypes, omeprazole inactivated CYP2C19 with similar K_I_ values of either 3.01 ± 0.83 µM for RMs, 4.47 ± 1.8 for NMs and 8.9 ± 12.38 µM for IMs. The mean maximal rate of inactivation (K_inact_) was 0.028 ± 0.002 min^−1^ for RMs, 0.031 ± 0.004 min^−1^ for NMs and 0.026 ± 0.01 min^−1^ for IMs, and not different between the genotype groups. Similar inactivation rate constants for CYP2C19 for omeprazole were reported by Shirasaka *et al.* in a microsome pool of 7 non-genotyped donors (Shirasaka et al., 2013). Altogether this suggest that the intrinsic inhibitory potency of omeprazole is not affected by the *CYP2C19* genotype.

**FIGURE 4:**
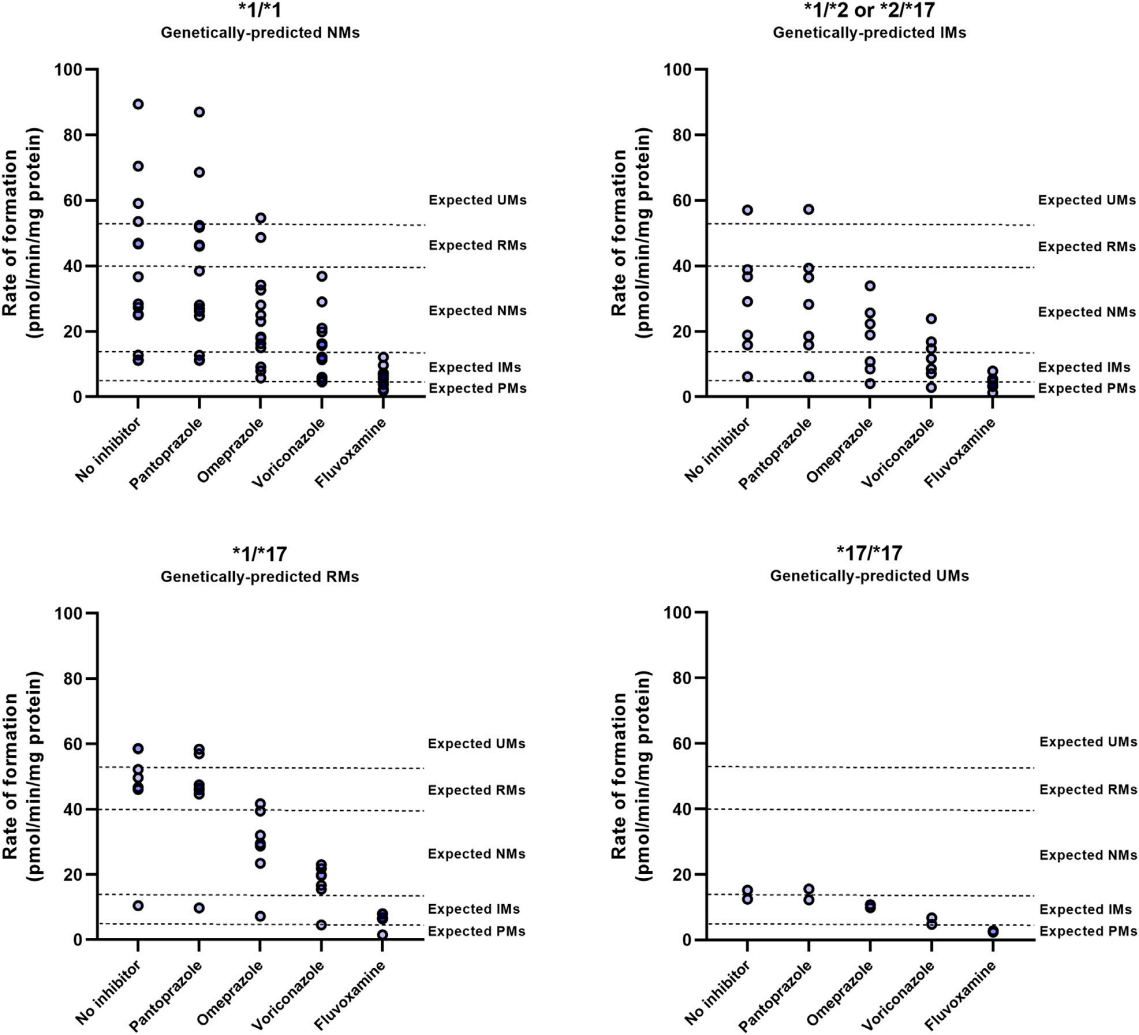
CYP2C19 inhibitor-induced phenoconversion of CYP2C19 metabolism in various CYP2C19 genotypes. Individual microsomal fractions were co-exposed to clinically relevant concentrations of inhibitors and residual CYP2C19 activity was measured. Concentrations resembled calculated unbound maximal hepatic inlet concentrations for either 100 mg fluvoxamine, 40 mg omeprazole, 200 mg voriconazole or 40 mg pantoprazole (standard dosing). Donors that were already phenotypically measured to be PM at baseline were excluded for treatment with inhibitors. Phenotype thresholds were based on Kiss et al. (Kiss et al., 2018), after applying a scaling factor for S-mephenytoin substate concentration used in this experiment.

To investigate whether genotype impacts the outcome of DDIs with a CYP2C19 inhibitor, individual microsomes were co-exposed to inhibitors and the observed phenotypic switch was classified (Figure 4; Supplementary Table S1). The consequences of CYP2C19 inhibitor-mediated phenoconversion were different between *CYP2C19* genotypes. In *1/*1 donors, voriconazole caused 50% of donors to exhibit residual activities representing IMs or lower, whereas only 14% of *1/*17 exhibited such activities. Of the genetically-predicted IMs, 5 out of 7 donors displayed NM activities at baseline. Subsequent voriconazole treatment resulted in 57% of genetically-predicted IMs to show a IM or PM phenotype. Likewise, although fluvoxamine converted all donors to phenotypic IMs or lower, predicted RMs (14%) were less likely to be converted to functional PMs than predicted NMs (50%) or IMs (57%). Treatment with omeprazole resulted in 43% of genetically-predicted IMs to exhibit IM or PM activities, whereas this was 21% for *1/*1 and only 14% for *1/*17 donors. The two donors with a *17/*17 genotype converted to either IMs or PMs upon inhibitor treatment, but this phenoconversion may be an overprediction due to low basal activity in these donors. Pantoprazole did not result in phenoconversion in any of the genotypes.

These results suggests that the differential outcomes of CYP2C19-mediated DDIs between genotypes are not dictated by distinctive inhibitory strengths between genotypes but by the donors basal CYP2C19 activity, that may in part be predicted by *CYP2C19* genotype.

## Discussion

In this study we aimed to quantify to what extent *CYP2C19* polymorphisms can impact the outcome of a DDI with various CYP2C19 inhibitors in human liver microsomes. In order to deliver recommendations for DDGIs it is imperative to acquire a quantitative comprehension of the phenoconversion that arises subsequent to the co-administration of an inhibitor targeting the same enzyme. Our results demonstrate that the outcome of a DDI is dictated by both inhibitor strength and CYP2C19 activity, which is in turn dependent on genotype and non-genetic factors including comorbidities. This study provides a quantitative understanding of the magnitude of DDGIs, which can ultimately aid in tailoring drug therapy recommendations to an individual’s needs.

Phenoconversion due to the use of concomitant medication can limit the accuracy of pharmacogenetic-based drug dosing. As such, considering concomitant medication use seems an integral part of *CYP2C19* pharmacogenetic-based personalized therapy. Quantitative data is required to assess phenoconversion after concomitant medication use. Mostafa *et al.* used a conservative approach to predict the corrected phenotype following the use of concomitant moderate or strong CYP2C19 inhibitors (Mostafa et al., 2019). They estimated that carriers of one or two functional alleles (*1) would convert to a PM, and carriers of one or two increased functional alleles (*17) would convert to an IM phenotype. Our results on strong inhibition are in accordance with these predictions. Fluvoxamine, a strong inhibitor of CYP2C19, caused 86% of *1/*17 donors to become phenotypically IM, whereas most of genetically-predicted IMs were converted to a PM phenotype (57%). In accordance with unaltered CYP2C19 activity in patients with gastroesophageal reflux disease taking pantoprazole, weak inhibition by pantoprazole did not induce phenoconversion (Modak et al., 2016).

However, the outcomes of DDIs with moderate inhibitors (omeprazole/voriconazole) matched less well to the proposed phenoconversion model by Mostafa *et al*, which predicted that NMs/IMs convert to a PM phenotype upon moderate inhibition of CYP2C19. In our study, voriconazole, which acts as a moderate CYP2C19 inhibitor, significantly reduced the drug metabolizing capabilities of CYP2C19 by approximately one level (i.e., from a phenotypic NM to a IM). As a result, 40% of the donors (12/30) were converted into IM or PM phenotypes by voriconazole. Though, none of the NMs were converted into PMs, except for one donor who already exhibited impaired CYP2C19 activity in the absence of voriconazole treatment (basal phenoconversion). For omeprazole, phenoconversion into IM or PM phenotypes was even less frequently seen, in only 10% of the donors (3/30). These findings are in contrast to a clinical study, in which the pantoprazole-^13^C breath test indicated that 96% of patients converted to a PM phenotype after treatment with omeprazole or esomeprazole (Klieber et al., 2015). The underlying cause of these significant alterations in the phenotype upon PPI treatment observed in this study remains unclear. Especially since concomitant administration of omeprazole generally results in changes in area under the curve (AUC) of low magnitude (<2-fold), with little clinical importance (Ogawa and Echizen, 2010). Moreover, a study on the effect of omeprazole on the pharmacokinetics of the CYP2C19 substrate moclobemide showed that the AUCs of NMs after omeprazole treatment did not reach the observed AUCs of PMs within the study, indicating phenoconversion to an IM rather than a PM phenotype (Yu et al., 2001). Altogether, our data suggest that CYP2C19 inhibition by moderate inhibitors can result in phenoconversion, but it seems unlikely to result into a PM phenotype for wild-type *1/*1 genotypes.

Omeprazole is considered to be a MDI indicating that part of its inhibitory activity of CYP2C19 is dependent on the biotransformation of omeprazole into its active metabolites. For this reason, we hypothesized that the inhibitory potency (K_I_/K_inact_) of omeprazole could be affected by the *CYP2C19* genotype. Nonetheless, our data in CYP2C19 genotype-matched donor pools showed no effect of *CYP2C19* genotype on the inhibitory potency of omeprazole. This is in accordance with results for paroxetine, a MDI of CYP2D6, for which the inhibitory parameters were also similar between different CYP2D6 genotypes in a microsomal assay (Storelli et al., 2019). These two studies highlight that the type of inhibitor (direct vs. MDI) is presumably not a determinant in the outcome of DDI-induced phenoconversion in donors with different genotypes. Instead, our study reinforces that the outcome of a DDI and the conversion of a patients phenotype depends on both the strength of the CYP2C19 inhibitor and the basal activity of CYP2C19. Therefore, both factors should be taken into account for phenotype predictions, as successfully demonstrated for CYP2D6 (Borges et al., 2010).

As mentioned, one primary factor in determining the outcome of a DDI is the initial enzyme activity, which is partly determined by an individual’s genotype. However, our cohort also revealed discordance between genotype-based prediction of CYP2C19 activity and actual metabolizing capacity at baseline. These marked genotype-phenotype discrepancies for CYP2C19 metabolism are consistent with other studies. In a large PK study, Lorenzini *et al.* reported the concordance between *CYP2C19* genotype-predicted phenotypes and measures phenotypes and showed a low(er) concordance for genetically-predicted NMs (33%) and UM’s (19%) in comparison to genetically predicted IM’s (91%) (Ing Lorenzini et al., 2021). This CYP2C19 genotype-phenotype discrepancy is retained in different ethnic populations (De Andrés et al., 2016; de Andrés et al., 2017; de Andrés et al., 2021). In isolated microsomes, Kiss *et al.* reported, similarly to our own results, a 40% concordance (Kiss et al., 2018). Importantly, we found a 2.5 fold increase in the occurrence of PMs among our donors than what would be expected based on genotype data. This is in concordance with previous population studies which report that the prevalence of phenotypic PMs could be up to 5–10 fold higher than genetically-predicted (Mostafa et al., 2019; Gloor et al., 2022). This could have important consequences, as drug interactions are typically pertinent when an individual has a poor or intermediate capacity in the primary metabolic pathway. Indeed, various clinical studies indicate that PMs are at risk of decreased responsiveness or toxicity during CYP2C19 substrate therapy (i.e., citalopram, omeprazole and clopidogrel) (Hicks et al., 2015; Lima et al., 2021; Lee et al., 2022). It is therefore crucial to consider factors that could be responsible for phenotype-genotype discrepancies and thereby evoke phenoconversion and phenotypic poor metabolism despite the presence of functional alleles.

A recent clinical phenotyping study by Gloor *et al.* demonstrated that concomitant medication use could only explain 32% of the CYP2C19-related phenoconversion (Gloor et al., 2022). This underscores the importance of non-genetic factors and presumably disease-related effects on CYP2C19 activity. In our cohort, the inclusion of disease-related information could provide an explanation why two RMs were phenotypically IMs/PMs, since even modest liver illness significantly affects CYP2C19’s ability to metabolize drugs (Frye et al., 2006). Another co-morbidity that is increasingly connected to changes in drug metabolism is diabetes mellitus (Darakjian et al., 2021; Neyshaburinezhad et al., 2023). In three of the four donors suffering from diabetes mellitus, a PM phenotype was observed despite the presence of one or two functional alleles. Importantly, the observed disease-related changes were not related to C-reactive protein (CRP) suggesting that metabolic rather than inflammatory mechanisms contribute to these disease-related changes in drug metabolism. Hence, similar to conclusions made by Kiss *et al.*, including disease-related factors could help to enhance the prediction of the CYP2C19 phenotype (Kiss et al., 2018).

There is an increased interest in finding biomarkers to predict the rate of drug metabolism in the liver to facilitate phenotype predictions (Rowland et al., 2019; Achour et al., 2022). We investigated whether mRNA expression in the liver itself can predict the hepatic metabolizing capacity of CYP2C19. As previously reported, total CYP2C19 mRNA levels were not a good predictor of CYP2C19 mRNA activity (Rodríguez-Antona et al., 2001; Pridgeon et al., 2022). One major limitation of expression studies is that the functional consequences of the produced mRNA are not taken into account when assessing the relationship between mRNA expression and activity. For example, with respect to CYP2C19, the CYP2C19*2 alleles are linked to splicing defects of mRNA and hence formation of inactive protein (Chaudhry et al., 2015). Therefore, to better examine the true relationship between mRNA expression and activity, we utilized a primer-pair that predominantly detects functional mRNA and not CYP2C19*2 mRNA. Examining functional CYP2C19 mRNA indeed improved the correlation between expression and activity by ∼2 fold, but a large proportion of the variance remained unexplained. Moreover, the moderate correlation that was observed was largely driven by the genetic PMs within our cohort. This reinforces that, in addition to genotyping, incorporation of hepatic mRNA expression provides limited complementary value for predicting the drug metabolizing capacity of individuals.

There are some limitations to address. First of all, the phenotype thresholds used to define phenoconversion are based on values reported in literature and might over- or overpredict the extent of phenoconversion. However, phenotype assessment is essential in order to ultimately create DDGI guidelines, since dosing adjustments are made based on phenotypes in clinical practice. Van der Lee *et al.* proposed that a patient’s phenotype prediction can be improved by using a continuous scale for this prediction rather than a set threshold between two phenotype groups (Van der Lee et al., 2021). Still, 21% of interindividual variability in CYP2D6 could not be explained by this approach, rendering it likely that non-genetic factors contribute to this variability. As such, the CYP450 genotype should be interpreted in the clinical context of the individual patient, considering all feasible contributors to CYP450 metabolic function. Borges *et al.* used a scoring system that incorporates both CYP2D6 genetic variation and CYP2D6 mediated DDIs, which showed to improve phenotype prediction as compared to genetic information alone (Borges et al., 2010). Such a scoring system lends itself well to be extended to other non-genetic factors, such as the presence of liver disease or other comorbidities. A scoring system tool that incorporates both CYP2C19 activity on a continuous scale, together with the inhibitory effect of DDIs and comorbidities (i.e., liver disease) will likely improve the pharmaco-genotype to phenotype translation.

Secondly, this study was conducted in liver biopsies that were genotyped for *2, *3 and *17 variants, as these alleles are most prevalent among Europeans and recommended for clinical testing by the pharmacogenetics working group of the American association for molecular pathology (Pratt et al., 2018). While disease-related factors may explain most of the observed phenoconversion into lower drug-metabolizing phenotypes among our patients, it is important to consider that other (rare) genetic variants within CYP2C19 could also have influenced the mismatch between predicted and observed activities in our study (Ingelman-Sundberg et al., 2018). Furthermore, it is necessary to acknowledge that extrapolating our findings to non-European populations may be challenging due to differences in the genomic architecture of CYP2C19 across populations (Zhou et al., 2017). Therefore, investigating phenoconversion in other populations, such as Asians or Africans, where alleles like *3 or *9 may contribute to basal activity and modulate DDIs for CYP2C19-dependent drugs, would be of great interest.

Another potential limitation relates to the selection of concentrations of the inhibitors in this study. Input parameters for calculating these concentrations were dependent on available literature. Still, the EMA and FDA support that the unbound maximum hepatic inlet concentration adequately mimics the clinical inhibition of hepatic P450 enzymes (Parkinson, 2019). Goutelle *et al.* utilized reported AUCs in NMs with and without CYP2C19 inhibitors, along with the contribution ratio of the substrate drug, to calculate inhibitory potencies of CYP2C19 inhibitors for predicting drug interactions *in-vivo* (Goutelle et al., 2013). Their calculated AUC ratios for omeprazole 40 mg/day and voriconazole 400 mg/day were 43% and 66%, which are consistent with the inhibitory potency observed in our microsomal assay (37% and 59%, respectively). It should be noted that our chosen concentration of fluvoxamine may underestimate the phenoconversion to some extent since we report 85% inhibition, whereas Goutelle *et al.* reported 97%. Calculated unbound maximum hepatic inlet concentrations used in our assay are thus likely to represent the observed inhibitory potencies *in-vivo*. A clinical trial investigating the risk of DDI-induced CYP2C19 phenoconversion in healthy volunteers is now ongoing, and will likely inform whether the magnitude of CYP2C19 inhibition observed in our *in-vitro* system matches a clinical setting (NCT05264142).

In conclusion, this study suggests that the differential outcomes of CYP2C19-mediated DDIs are not determined by different inhibitory strengths between genotypes, but by the basal activity of CYP2C19. This activity can in part be predicted by *CYP2C19* genotype, but is also influenced by disease-related factors. This underlines the necessity to integrate both genetic data as well as comedication use and disease-related factors into a person’s predicted phenotype.

## Data Availability

The original contributions presented in the study are included in the article/Supplementary Materials, further inquiries can be directed to the corresponding author.
