# Supplementary material for: The impact of CYP2C19 genotype on phenoconversion by concomitant medication
Source: Front Pharmacol. 2023 Jun 8;14:1201906. doi: 10.3389/fphar.2023.1201906 (PMC10285291; doi:10.3389/fphar.2023.1201906)
Supplement: Supplementary file 1 [file Table1.docx]

Supplementary Material

## Supplementary Figures

**
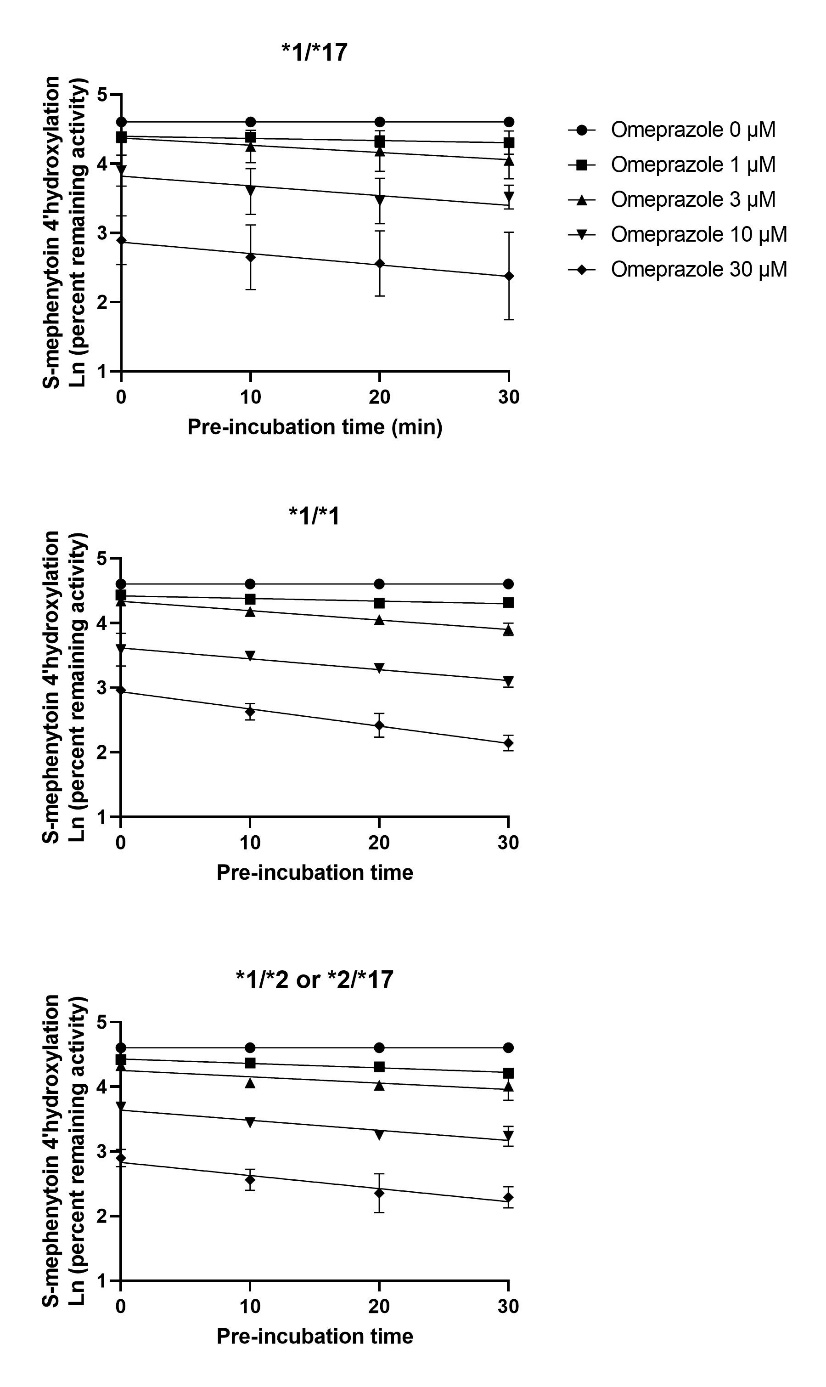
**

**Supplementary Figure S1.** Time dependent inhibition of CYP2C19 at various concentration of omeprazole. Omeprazole was pre-incubated for 0-30 minutes at concentrations 0-30 µM and residual CYP2C19 activity was measured, see materials & methods “*Kinetic analysis of CYP2C19 dependent S-mephenytoin hydroxylation*”. The slope of each line is the value of the observed rate constant (K_obs_) for the inactivation of CYP2C19 by omeprazole at a given concentration. Individual points represent the average of triplicate determinations ± SD

**
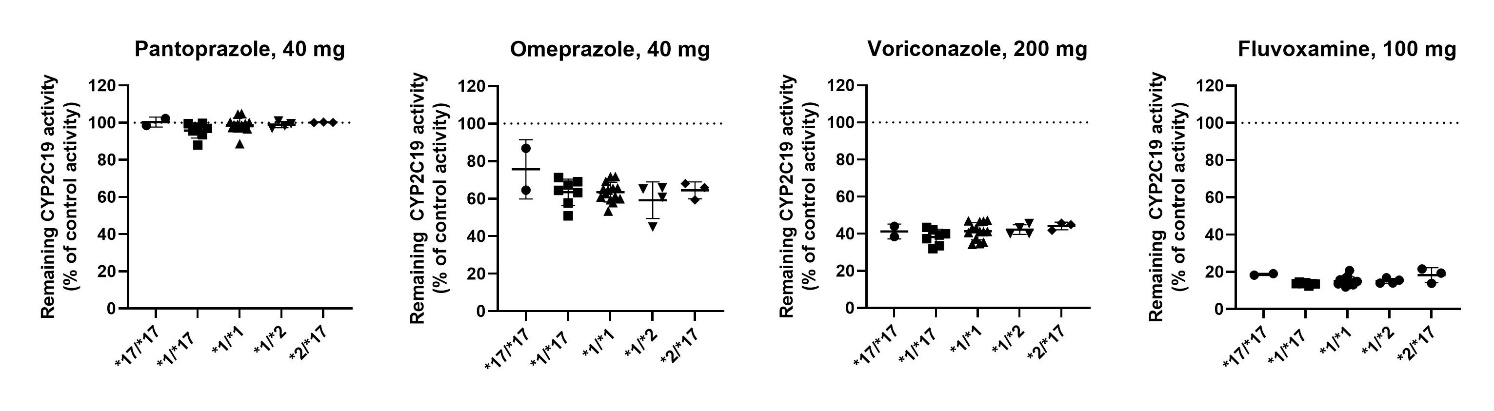
**

**Supplementary Figure S2.** Decreased activity of CYP2C19 following inhibitor treatment is independent of genotype. For every inhibitor and genotype, S-mephenytoin 4’-hydroxylation activity is shown as compared to control (no inhibitor, 100%). A one-way ANOVA with Dunnett’s post hoc test was done to test whether the percentual decrease was different between genotypes.

**Supplementary Tables**

Supplementary Table S1. Remaining phenotype after treatment with various CYP2C19 inhibitors for different genotype groups.

|  | **No inhibitor (n=40*)** | **Pantoprazole (n=30)** | **Omeprazole (n=30)** | **Voriconazole (n=30)** | **Fluvoxamine (n=30)** |
| --- | --- | --- | --- | --- | --- |
| *17/*17 (genetically predicted UMs) | 1× NM  1× IM | 1× NM (50%)  1× IM (50%) | 2× IM (100%) | 1× IM (50%)  1× PM (50%) | 2× PM (100%) |
| *1/*17 (genetically predicted RMs) | 2× UM  4× RM  1× IM ---------  *1*× *PM* | 2× UM (29%)  4× RM (57%)  1× IM (14%) | 1× RM (14%)  5× NM (71%)  1× IM (14%) | 6× NM (86%)  1× PM (14%) | 6× IM (86%)  1× PM (14%) |
| *1/*1 (genetically predicted NMs) | 4× UM  2× RM  5× NM  3× IM ---------  *2*× *PM* | 2× UM (14%)  4× RM (29%)  5× NM (35%)  3× IM (21%) | 1× UM (7%)  1× RM (7%)  9× NM (64%)  3× IM (21%) | 7× NM (50%)  6× IM (43%)  1× PM (7%) | 7× IM (50%)  7× PM (50%) |
| *1/*2 or *2/*17 (genetically predicted IMs) | 1× UM  5× NM  1× IM ---------  *3*× *PM* | 1× UM (14%)  5× NM (71%)  1× IM (14%) | 4× NM (57%)  2× IM (29%)  1× PM (14%) | 3× NM (43%)  3× IM (43%)  1× PM (14%) | 3× IM (43%)  4× PM (57%) |
| *2/*2 (genetically predicted PMs) | *4*× *PM* |  | | | |

* Donors (indicated in *italics, n=10)* that were phenotypically measured to be PM at baseline were excluded for treatment with inhibitors. Percentages indicate phenoconverted individuals per genotype group.
