# Supplementary material for: The impact of CYP2C19 genotype on phenoconversion by concomitant medication
Source: Front Pharmacol. 2023 Jun 8;14:1201906. doi: 10.3389/fphar.2023.1201906 (PMC10285291; doi:10.3389/fphar.2023.1201906)
Supplement: Supplementary file 2 [file Table2.docx]

Supplementary Material

**Supplementary material & methods**

Primer sequences and amplification efficiencies

Supplementary Table S1. Primer sequences and amplification efficiencies. Amplification efficiency (%) was calculated using the formula: $\left( {10}^{\frac{-1}{slope}}-1 \right)*100$.

|  | **Sequence** | **Amplification efficiency** |
| --- | --- | --- |
| CYP2C19 *functional* | For 5’-AAAACCAAGGCTTCACCCTGTGATCC-3’ Rev 5’-CCGGGAAATAATCAATGATAGTGGGAAA-3’ | 98.7% |
| CYP2C19 *total* | For 5’-GCTCTCTTTCCTCTGGTCCAAATTTCAC-3’ Rev 5’- GCACAGTGAAACTTTTTTAATGGAGGCTG-3’ | 99.2% |
| CRP | For 5’-CTCTCTCATGCTTTTGGCCAGACAG-3’ Rev 5’-AAGAATTCACAGCCCCACAAGGTTC-3’ | 96.3% |
| PNLPA3 | For 5’-TCACTCGAGTGCTGATGTGTCTGC-3’ Rev 5’-CCTCTGCTTTGGTCTCTGCTGGAC-3’ | 97.8% |

Quantification of 4’hydroxymephenytoin by LC-MS/MS

Quantification of 4’hydroxymephentoin in the microsomal incubations was done using a liquid chromatography-tandem mass spectrometry (LC-MS/MS) system consisting of a Nexera LC-40 high-performance liquid chromatography (HPLC) system equipped with a DGU-403 degassing unit, two LC-40D pumps, a SIL-40C autosampler, and a CTO-40S column oven (Shimadzu, ‘s-Hertogenbosch, the Netherlands). A Kinetex C18 column (1.7 µM, 50x2.1 mm) (Phenomenex, Utrecht, The Netherlands) with a SecurityGuard Ultra C8, 2.7 µm, 5 × 2.1 mm cartridge (Phenomenex, Utrecht, The Netherlands) as guard column were used to separate 4’hydroxymephenytoin from other analytes present in the sample matrix. Mobile phases consisted of water (A) and methanol (B) both containing 0.1% formic acid. The gradient, with a flow rate of 0.4 ml/min, started at 5% B and increased to 100% B in 4 min, maintaining 100% B for 2 min, and then returned to initial conditions for another 2 min. The column was kept at 50 ˚C and the injection volume was 20 µL. The HPLC was coupled to a Sciex QTRAP 6500+ mass spectrometer (AB Sciex Netherlands B.V., Nieuwerkerk aan den Ijssel, The Netherlands) operating in positive electrospray mode (ESI+).

The MS conditions were as follows: curtain gas 20 psi, collision gas “medium”, ion source gas 1 40 psi, ion source gas 2 40 psi, ion spray voltage 5500 V and temperature 550 ˚C. The MS was operated in the multiple reaction monitoring (MRM) mode and was optimized by direct infusion of the standards individually. The optimized MRM transitions, retention time, declustering potential (DP), collision energy (CE) and cell exit potential (CXP) for 4’hydroxymephenytoin and internal standard 4’hydroxymephenytoin-d_3_ are summarized in Supplementary Table 2.

Supplementary Table S2. MRM parameters and retention time for the quantified analytes by the LC-MS/MS method.

| **Analyte** | **Q1 mass (Da)** | **Q3 mass (Da)** | **Retention time (min)** | **DP (V)** | **CE (V)** | **CXP (V)** |
| --- | --- | --- | --- | --- | --- | --- |
| 4’-hydroxymephenytoin | 235.1 | 150.1 | 2.7 | 51 | 25 | 10 |
| 4’-hydroxymephenytoin-d_3_ | 238.1 | 150.1 | 2.7 | 41 | 25 | 14 |

Assay accuracy and precision were determined by analyzing quintuplicates of quality controls at five concentration levels quality controls that were prepared like the microsomal samples. Within – and between runs coefficients of variation (CV) were ≤2% (n=3). The mean bias was in the range of -4% to 7% across all concentration levels (n=3). Analyst software version 1.4 (AB Sciex Netherlands B.V., Nieuwerkerk aan den Ijssel, The Netherlands) was used for data analysis.

Calculating the unbound maximum hepatic inlet concentration

The unbound maximum hepatic inlet concentration in plasma incorporates the sum of two concentrations, namely the maximum concentration of drug in plasma (Plasma I_max_) and the maximum concentration of drug that was absorbed from the gut into the hepatic portal system (Total portal C_max_ in plasma*)*, and is predicted to adequately mimic the clinical inhibition of hepatic P450 enzymes (1).

The mean maximum concentration of inhibitors in plasma after dosing to steady state (Plasma I_max_*)* with the chosen clinical dose was retrieved from literature (Supplementary Table 3).

Supplementary Table S3. Retrieved mean total systemic I_max_ values in plasma for clinically relevant dosages of CYP2C19 inhibitors.

|  | Dose (mg) | Mean Plasma I_max_ (µM) | References |
| --- | --- | --- | --- |
| Fluvoxamine | 100 | 0.3 | Summarized from references within (2) |
| Omeprazole | 40 | 3.3 | (3–6) |
| Voriconazole | 200 | 7.3 | Summarized from references within (7) |
| Pantoprazole | 40 | 6.5 | (8) |

**Supplementary method references**

1. Parkinson A. Regulatory Recommendations for Calculating the Unbound Maximum Hepatic Inlet Concentration: A Complicated Story with a Surprising and Happy Ending. Drug Metab Dispos [Internet]. 2019 Jul 1 [cited 2022 Nov 3];47(7):779–84. Available from: https://pubmed.ncbi.nlm.nih.gov/31048452/

2. Britz H, Hanke N, Volz AK, Spigset O, Schwab M, Eissing T, et al. Physiologically-Based Pharmacokinetic Models for CYP1A2 Drug-Drug Interaction Prediction: A Modeling Network of Fluvoxamine, Theophylline, Caffeine, Rifampicin, and Midazolam. CPT pharmacometrics Syst Pharmacol [Internet]. 2019 May 1 [cited 2023 Feb 26];8(5):296–307. Available from: https://pubmed.ncbi.nlm.nih.gov/30762305/

3. Braeckman RA, Stirtan WG, Soni PN. Effect of Icosapent Ethyl (Eicosapentaenoic Acid Ethyl Ester) on Omeprazole Plasma Pharmacokinetics in Healthy Adults. Drugs R D [Internet]. 2014 Sep 1 [cited 2023 Feb 26];14(3):159. Available from: /pmc/articles/PMC4153963/

4. Hassan-Alin M, Andersson T, Niazi M, Röhss K. A pharmacokinetic study comparing single and repeated oral doses of 20 mg and 40 mg omeprazole and its two optical isomers, S-omeprazole (esomeprazole) and R-omeprazole, in healthy subjects. Eur J Clin Pharmacol [Internet]. 2005 Jan [cited 2023 Feb 26];60(11):779–84. Available from: https://pubmed.ncbi.nlm.nih.gov/15578172/

5. Roh HK, Kim PS, Lee DH, Tybring G, Sagar M, Park CS, et al. Omeprazole Treatment of Korean Patients: Effects on Gastric pH and Gastrin Release in Relation to CYP2C19 Geno- and Phenotypes. Basic Clin Pharmacol Toxicol [Internet]. 2004 Sep 1 [cited 2023 Feb 26];95(3):112–9. Available from: https://onlinelibrary.wiley.com/doi/full/10.1111/j.1742-7843.2004.950302.x

6. Prichard PJ, Yeomans ND, Mihaly GW, Jones DB, Buckle PJ, Smallwood RA, et al. Omeprazole: a study of its inhibition of gastric pH and oral pharmacokinetics after morning or evening dosage. Gastroenterology [Internet]. 1985 [cited 2023 Feb 26];88(1 Pt 1):64–9. Available from: https://pubmed.ncbi.nlm.nih.gov/3880557/

7. Li X, Frechen S, Moj D, Lehr T, Taubert M, Hsin C hsuan, et al. A Physiologically Based Pharmacokinetic Model of Voriconazole Integrating Time-Dependent Inhibition of CYP3A4, Genetic Polymorphisms of CYP2C19 and Predictions of Drug-Drug Interactions. Clin Pharmacokinet [Internet]. 2020 Jun 1 [cited 2023 Feb 26];59(6):781–808. Available from: https://pubmed.ncbi.nlm.nih.gov/31853755/

8. Shao JG, Jiang W, Li KQ, Lu JR, Sun YY. Blood concentration of pantoprazole sodium is significantly high in hepatogenic peptic ulcer patients, especially those with a poor CYP2C19 metabolism. J Dig Dis [Internet]. 2009 [cited 2023 Feb 26];10(1):55–60. Available from: https://pubmed.ncbi.nlm.nih.gov/19236548/
